# A biological perspective towards a standard for information exchange and reporting in XAS

**DOI:** 10.1107/S1600577518008779

**Published:** 2018-06-27

**Authors:** Ritimukta Sarangi

**Affiliations:** a Structural Molecular Biology, Stanford Synchrotron Radiation Lightsource, SLAC National Accelerator Laboratory, 2575 Sand Hill Road, Menlo Park, CA 94306, USA

**Keywords:** biological XAS, EXAFS analysis, data reporting standard

## Abstract

The complex structural landscape of biological samples and their sensitivity to X-ray exposure leads to specific challenges in biological X-ray absorption spectroscopy (bio-XAS) research, which in turn has necessitated standardization of various aspects of bio-XAS data measurement, analysis and interpretation. The bio-XAS community is therefore well suited for the development of a data-reporting standard with the specific aim of creating a feedback loop for improving/standardizing data analysis protocols and optionally to make published data available to collaborators/researchers in a meaningful and quantitative format. The XIF (XAFS information file) reporting format presented here contains key experimental and analysis parameters, useful in developing a consistent platform for bio-XAS research worldwide. Such a reporting standard, enforced by the user community and publishing groups alike, can be an important step towards the standardization of data measurement and analysis techniques in bio-XAS.

## Abstract 2

An X-ray absorption spectroscopy (XAS) data-reporting format is developed and its inclusion as standard supporting information in biological XAS publications is proposed. The aim of this text-based data-reporting XIF (XAFS information file) file format is to develop a consistent protocol for XAS data collection, analysis and interpretation, and, optionally, for sharing reference and reported data.

## 1. Introduction   

The advent of third-generation synchrotron sources has paved the way for an impressive growth in the application of synchrotron X-ray absorption spectroscopy (XAS) in all areas of scientific enquiry. In particular, biological/biomimetic systems have gained significantly from the improvement in flux and beam sizes. The technique has developed dramatically since the first EXAFS experiment for local-structure determination in a biological system was performed on the non-heme iron protein rubredoxin by Shulman *et al.* (1975 ▸), who used Fe *K*-edge EXAFS to study its [FeS_4_]-containing active site. Shulman *et al.* not only obtained accurate bond distances for the active site but also showed that the active-site crystallographic structures were quantitatively incorrect. This study and follow-up publications (Eisenberger *et al.*, 1978 ▸; Shulman *et al.*, 1978 ▸) were instrumental in elevating EXAFS not only as the better technique for structurally probing metal sites in biology but also one that could improve the accuracy of crystallography work. Starting from this study in 1975, bio-XAS has made strong fundamental contributions towards research that hinged on the understanding of transition-metal-containing biological systems and has been instrumental in developing and extending the technique beyond bio-EXAFS, to the study of a wide range of molecular systems involved in biomimetic and homogeneous catalysis.

The EXAFS data for rubredoxin were evaluated using the simplified Stern–Sayers–Lytle single-scattering equation
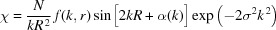
to determine the Fe—S distances. Shulman *et al.* used a model compound composed of the same central atom and the same ligand where the Fe—S distances (*R*) were known (and equal, within the experimental accuracy). The absorption of the model compound was used to determine α(*k*) as a function of *k* and these values, in conjunction with the absorption data of rubredoxin, were used to determine its distances. This approach of using a reference compound to extract phase parameters gave way to more rigorous theoretical treatments in the 1980s, which became available in the easy-to-use theoretical packages *GNXAS* (Filipponi & Di Cicco, 1995 ▸; Filipponi *et al.*, 1995 ▸), *FEFF* (Rehr *et al.*, 1991 ▸) and *EXCURVE* (Tomić *et al.*, 2004 ▸), spurring the interest of synchrotron non-experts. Early impact on biological EXAFS was made by *EXCURVE*, which provided an integrated and user-friendly approach, while simultaneously contributing to theory development (spherical-wave method, multiple-scattering theory, *etc*.) (Gurman *et al.*, 1984 ▸, 1986 ▸). Of the three packages, *FEFF* has seen the widest adoption. For bio-EXAFS application two front-end codes, *EXAFSPAK* (George, 2000 ▸) and *GNXAS* (Filipponi & Di Cicco, 1995 ▸; Filipponi *et al.*, 1995 ▸), were among the earlier ones available to the general non-expert user. Some of the most important bio-EXAFS discoveries were made using this combination of theoretical packages and platforms. A biologically relevant feature of *EXCURVE* is the constraint/restraint model of data fitting, which can be coupled with structural models obtained from protein crystallography to restrict the structure refinement and exclude both chemically impossible solutions and those that were not supported by protein crystallography (Binsted *et al.*, 1992 ▸).

It is important to emphasize that the theoretical development of EXAFS was happening at pace with the adoption of EXAFS by the non-expert research community. For example, the *FEFF* code has continually evolved over the last two decades and the modern *FEFF9* code is theoretically advanced over that used in the early 1990s. This simultaneous development of theory and its adoption by the non-expert user, fueled by the fact that EXAFS data analysis, for the most part, is a trial-and-error process of testing theoretically calculated parameters against the experimental data, led to the development of ‘personalized’ data-fitting protocols by research groups around the world. Early adopters of EXAFS had their own data-fitting protocols, which, although strongly rooted in theory, thwarted standardization of data-fitting and analysis techniques. Today, EXAFS data are analyzed using a variety of different data-fitting packages, some of which are ‘home-brewed’ and ‘personalized’ data-fitting guidelines followed to varying levels of rigor.

The early success of synchrotron-based X-ray spectroscopy and EXAFS has also led to strong investment in beamlines around the world (see the IXAS portal, https://www.ixasportal.net/ixas/index.php). Most synchrotron facilities today have at least one EXAFS beamline and the large increase in the number of synchrotron facilities means that there are upwards of a hundred EXAFS-capable beamlines around the world, built on bending magnets to insertion devices, such as wigglers and undulators. In addition, EXAFS beamlines operate extremely customized instrumentation, designed with specific objectives, such as alignment with the synchrotron source parameters, compatibility with one or more experimental capabilities supported by the beamline, capability to support the desired flux, timing and spot size *etc*. EXAFS beamlines also have customized detector systems and sample environments to cater to the various science areas they support. The fallout of this diversity in beamlines and experiment-specific instrumentation is the observed quantifiable spectral difference affecting not only the data analysis techniques followed by the researcher but the actual information content of the XAS data.

The rapid growth of XAS and its widespread adoption, along with simultaneous theory development and customization in beamline development, has shaped modern biological EXAFS data analysis and interpretation. Here, some of the challenges posed by biological systems are listed and in light of these challenges a data-reporting standard is proposed, with the aim of serving the needs of the EXAFS researcher and to facilitate the researcher in sharing and understanding data from other researchers and user facilities.

## 2. Biological XAS and EXAFS   

Biological systems present some of the most challenging problems for XAS applications. They can be notoriously sensitive to manipulation and are susceptible to fast photoreduction and photodamage in the X-ray beam, attributed largely to the aqueous substrate, which forms hydroxide radicals in the X-ray beam (George *et al.*, 2012 ▸). These radicals non-specifically attack the protein matrix and are instrumental in reducing redox-active metal centers. Bio-XAS experiments are performed under low-temperature conditions, preferably under liquid He, to ameliorate X-ray beam related damage. With the advent of high-flux-density beamlines, such beam damage is becoming increasing difficult to manage. One of the many approaches available today for continually supplying fresh sample for exposure and measurement (such as raster or point-to-point scanning over a fixed frozen sample, liquid-jet delivery, continuous sample flow system) has become essential. On the other hand, biological samples are typically available in sparse quantities (hundreds of µL) and at low concentrations (0.1–10 m*M*), which severely limits these high sample demand procedures used to minimize/avoid beam damage. Bio-EXAFS measurements can also be challenging due to ice-diffraction by the frozen microcrystalline water molecules present during low-temperature measurements. Ice-diffraction can affect several detector elements in a multi-element solid-state detector system, making the detector less efficient and necessitating longer collection times for high-quality data. This can be addressed by adding glassing agents such a glycerol or glycol to the protein samples, which, upon rapid freezing in liquid nitro­gen, result in an ice-free glass. However, these glassing agents reduce the effective concentration of the sample (∼30–40% glycerol by volume is required to form a good glass) and in some cases facilitate fast photoreduction by generating more hydroxide radicals.

### 2.1. Architecture of metals in biological systems   

Metals play life-essential roles in biological processes and are ubiquitously required for the survival of organisms from all walks of life. In particular, *d*-block elements, including all first-row elements, Mo and W play crucial catalytic roles as the central metal ion in the active sites of metalloproteins. Nature has beautifully adapted these elements into active sites by providing a wide array of both endogenous and exogenous ligand systems, which manage active-site reactivity by tuning its electronic structure, redox potentials, pK_a_, steric environment, dielectric properties, *etc*. These metal ions are seamlessly integrated into the protein matrix but are typically not irreversibly bound. They are designed to be flexible and to accommodate changes in coordination numbers, site-geometry and redox states during catalysis, while the protein matrix, which is in an aqueous solution, subtly changes around it to facilitate function.

**Figure 1:**
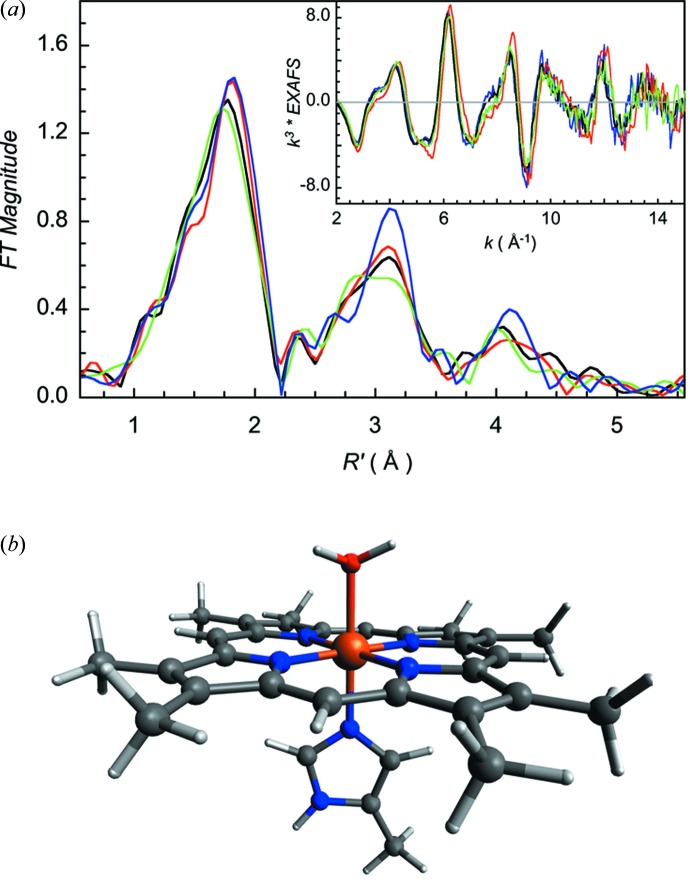
(*a*) Comparison of the Fe *K*-edge EXAFS data (inset) and their corresponding Fourier transforms for resting ferric forms of hemoglobin (black line) (Wilson *et al.*, 2013 ▸), MauG (a heme-containing methyl­amine utilization protein) (red line) (Jensen *et al.*, 2012 ▸), CCP (cytochrome *c* peroxidase) (blue line) (Jensen *et al.*, 2012 ▸) and HO2 (heme oxygenase-2). (*b*) Structural schematic of the active site. The structures differ in the peripheral *R* groups, which are located ∼5 Å from the Fe center and have negligible contribution to the EXAFS data. These *R* groups have been truncated to methyl groups for simplicity of presentation.

This means that markedly different EXAFS spectra (especially in the second- and third-shell regions, which are dominated by multiple-scattering contributions) can be obtained from seemingly identical metal centers. A good example is the Fe *K*-edge EXAFS of heme sites in proteins. The central porphyrin in a heme is a heterocyclic, multidentate, planar ligand, which binds tightly to the metal center in a square-planar arrangement. The metal center often has one or two axial ligands (protein, solvent or external ligand based). The EXAFS of such systems is dominated by multiple-scattering contributions from the heme plane. Fig. 1(*a*) ▸ shows a comparison of Fe *K*-edge EXAFS and their corresponding Fourier transforms for four protein samples, each of which has the ‘identical’ [Fe^III^(H_2_O)(His)(heme)] active site (Fig. 1*b* ▸).

As is evident, there are small but real differences in the first-shell (Fe–N/O contribution) and larger, more dramatic, differences in the second-shell EXAFS region. These differences in the EXAFS spectra of seemingly identical metalloprotein active sites reflect the structural flexibility of metal sites in biology. Since the heme group is one of the most ordered/tightly bound sites in biology, other less ordered ‘identical’ active sites can reveal larger differences in the outer-shell EXAFS data. The structural flexibility of biological metal–ligand bonds illustrated in Fig. 1 ▸ has been systematically studied by Harding (2001 ▸, 2004 ▸) who compiled metal–ligand distances and angles from crystallographic data from small-molecules structures with biologically relevant metal–ligand bonds and from metalloproteins and their survey demonstrates the diversity of architecture in metal–ligand coordination.

In addition to life-essential metals, exogenous metals can also play a critical role in biology as therapeutic agents, medical contrast agents (Pt-based anticancer drugs or Gd-based MRI contrast agents). Metals also have toxic effects (such as Hg, Pb and As compounds) on life. Bio-XAS and EXAFS techniques plays an important role in elucidating the interaction of these exogenous metals with biological molecules and systems.

### 2.2. The ligand space of metallo-biomolecules   

Metal sites in biology are elaborate inorganic complexes (Holm *et al.*, 1996 ▸). Proteins coordinate metal sites with both endogenous and exogenous ligands. The endogenous ligands derive from nitro­gen, oxygen and sulfur coordination to the metal site and can be amino, amido, amidato, carbonyl or carboxyl­ate groups present on protein backbone or side-chains or exclusively side-chain-based ligands such as the imidazole (histidine), thiol­ate (cysteine), thio­ether (me­thio­nine), guanidine (arginine), phenolate (tyrosine), hydroxyl (serine, threonine) or di­sulfide ­(cystine). Some of these ligands can bind in mono- or bi-dentate fashion. Selenium-containing amino acids (seleno­cystine and seleno­methio­nine) are important ligand systems especially for bio-XAS since Se *K*-edge XAS and EXAFS measurements can furnish complementary structural and electronic information about the metalloprotein active site. This is also true of halogen ligands, especially bromide and iodide present in bio-molecules. Proteins contain macrocyclic prosthetic groups such as porphyrins, corrins, corphins and chlorins, which are covalently bonded to the protein backbone and strongly coordinate to metal centers. The biological ligand space also includes cofactors such as molybdopterins and α-ketoglutarate. A wide range of exogenous ligands are known to bind to metal-containing active sites, typically carbon-, oxygen- or nitro­gen-based, the most ubiquitous of these being water, hydroxide, oxide and sulfide. Organometallic complexes with CO, CN and alkyl groups (methyl, adenosyl, *etc*.) are present either in their resting state or transiently form during catalysis by the active-site metal ions. Metal ions also coordinate to nucleobases and other suitable binding sites in DNA and RNA. Finally, more complex exogenous ligands are derived from specific substrate interaction and binding to the metal sites. This diverse and complex landscape of ligands can lead to rich second- and third-shell contributions. Although the coordination of at least the endogenous ligands is constrained by the protein environment, the ligand can still have several different binding modes to the metal center, with angular variability of approach and binding, which has a profound effect on the EXAFS data.

### 2.3. Multimetallic clusters in biology   

**Figure 2:**
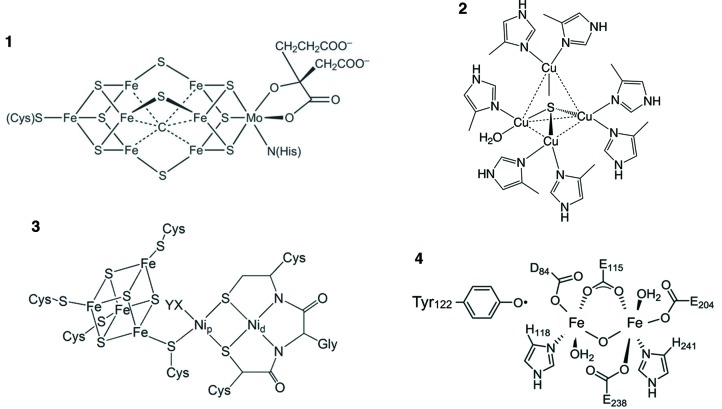
The metallocluster active sites in nitro­genase (FeMoCo) (**1**), nitrite reductase (Cu_z_) (**2**), acetyl CoA synthase (ACS) (**3**) and methane monooxygenase (MMO) (**4**). These sites illustrate the complexity of metal clusters found routinely in biological macromolecules.

Metalloprotein active sites catalyze energetically demanding reactions efficiently and under physiological conditions. To achieve this, transition-metal centers undergo dramatic electronic structure transformations, with large change in site geometry, oxidation states and spin states. This flexibility, unique to transition metals, has been masterfully utilized in multimetallic clusters, in which several metal centers work cooperatively in achieving significantly higher tunability of the electronic structure, minimization of reorganization energies and providing a robust and precise modulation of reactivity than can be achieved with a single metal center. For example, in the oxygen-evolving complex in photosystem II, the site of water oxidation has a Mn_4_OCa cluster at its active site (Vinyard *et al.*, 2013 ▸). This cluster undergoes a four-electron oxidation of its Mn_4_OCa-containing active site during its catalytic cycle (Yano & Yachandra, 2014 ▸). The actual oxidation state of each individual Mn center is hotly debated, but spectroscopic evidence clearly shows electronic coupling between the four Mn centers, which work in concert to effect the physiological oxidation of water to O_2_. Such multimetallic clusters are present at the hearts of several metalloproteins (examples are shown in Fig. 2 ▸) and have inspired bioinorganic synthetic research and bio-EXAFS characterization of the resulting biomimetic complexes. Biological/biomimetic metal clusters are unprecedented in nature, often with non-standard metal–ligand bond distances making the trial-and-error EXAFS fitting process difficult. Additionally, since the EXAFS data for homometallic clusters give an average over all probed metal atoms, structural information from individual metal centers is difficult to distinguish or characterize.

## 3. Modern approach to biological EXAFS   

### 3.1. Combined XANES and EXAFS investigation   

Despite the complex structural landscape of metals in biomolecules, EXAFS has made seminal contributions in the study of metalloprotein active sites. Early bio-XAS work by the research groups of R. Shulman, K. Hodgson, S. Hasnain, J. Penner-Hahn, S. Cramer, J. Dawson, R. Scott and others revealed structural insights that went far beyond the state-of-the-art in X-ray diffraction measurements at the time and galvanized the importance of EXAFS in studying metal sites in biology. In the last decade, applications of EXAFS have expanded to increasingly complicated systems and the scientific questions being addressed with EXAFS are pushing the limits of EXAFS analysis. These questions include angular information, multiple-scattering analysis of ligand systems to obtain three-dimensional information and attempts at teasing out small differences between like-atom ligands. At the same time, with theoretical advancement, the XANES region has become important to both assist and bolster assignments made from EXAFS analysis and as a stand-alone electronic structure determination technique. Developments in codes that allow the validity of a structural model to be judged against experimental data, such as the multiple-scattering-based *MXAN* (Benfatto *et al.*, 2001 ▸) and *FEFF9* (Rehr *et al.*, 2010 ▸) and the density functional theory (DFT) based *StoBe* (K. Hermann & L. G. M. Pettersson, http://www.fhi-berlin.mpg.de/KHsoftware/StoBe/), *Wien2k* (Blaha *et al.*, 1990 ▸), *NWChem* (Valiev *et al.*, 2010 ▸), *etc.* for XANES-based structure investigations have proven to be useful, especially on systems where EXAFS is not possible or in conjuction with EXAFS data, since the two techniques have orthogonal sets of advantages and disadvantages. These theoretical codes use an input structural model to either simulate the XANES spectra (up to ∼150 eV above the absorption edge) for comparison with experimental data or perform structural refinement to arrive at the structural model, which furnishes a XANES spectrum in good agreeement with experiment. For bio-XAS, the biggest impact has been made by DFT-based methods, that can simultaneously yield electronic and geometric structure information on a suitably truncated metal-containing active site. DFT-based quantum chemical packages simulate the spectral features arising from bound state electronic transitions (such as transition metal and ligand *K*-pre-edges and transition metal *L*-edges). In combination with time-dependent DFT (TD-DFT) methods, which simulate and assist in interpreting the metal *K*-pre-edge transitions (in first-row transition metal systems), DFT calculations brought about a holistic approach to bio-XAS, where the electronic structure obtained from the XANES region was interpreted by DFT methods and the corresponding structures obtained from the ‘spectroscopically calibrated’ theory were used as inputs for generating theoretical phase and amplitude parameters for EXAFS analysis. The quantum chemical packages *ORCA* (Neese, 2012 ▸) and *Gaussian* (Frisch, 2016 ▸) have been the frontrunners in such applications.

**Figure 3:**
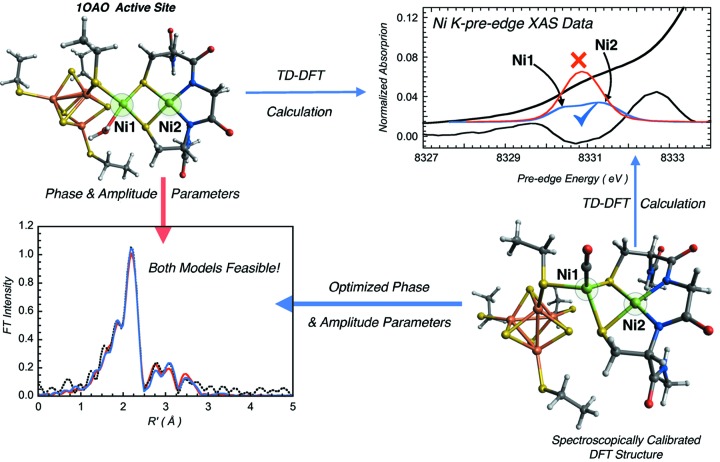
Schematic showing a comparison of Fourier transforms of EXAFS fits performed using phase and amplitude parameters calculated from the resting crystal structure (red) and the DFT-calculated structure (blue). Both structures reasonably fit the EXAFS data. The Ni *K*-pre-edge DFT simulation successfully differentiates between the incorrect (crystal structure) and the final optimized CO-bound models.

A recent study on the binuclear Ni active site in acetyl CoA synthase emphasizes the role of DFT in assisting EXAFS-based structure determination for comprehensive understanding of metal-containing active sites. In this study, the binding of CO to one Ni center in a binuclear Ni site hinged on evidence obtained from the DFT-calculated pre-edge transition that differentiated two structural models, both of which produced similar EXAFS fits. Without the electronic structure insights obtained from DFT calculations, the smaller contribution of one Ni—C bond in the complex active site of acetyl CoA synthase would be difficult to decipher and impossible to quantitate (see Fig. 3 ▸). Such DFT-assisted methods are fast becoming the standard in bio-XAS applications and the combination is being widely used to solve problems in biology by exploiting XAS’s unique ability to provide both geometric and electronic structure information on metal-containing sites, a feat unprecedented among spectroscopic techniques.

In recent years, bio-XAS researchers have taken advantage of X-ray spectrometer-based high-energy-resolution fluorescence-detected (HERFD) XAS measurements to obtain XAS and EXAFS data with higher energy resolution since the energy resolution of the spectrometer is of the order of the core-hole lifetime broadening. This also allows for ‘nearly’ background-free measurement and significantly enhances the signal-to-noise ratio of the spectrometer-based HERFD EXAFS experiment. For a typical biological system, where the elastic scattering and/or total background feature is significantly more intense than the fluorescence line of interest, the advantage of background removal (*i.e.* HERFD EXAFS) outweighs the higher total signal from the standard EXAFS measurement. The advantages of HERFD XAS have been well illustrated in recent studies of high-valent Fe-containing systems (Castillo *et al.*, 2017 ▸), Mo *K*-edge XAS in nitro­genase (Bjornsson *et al.*, 2014 ▸), the binuclear Fe center in hydrogenase (Mebs *et al.*, 2018 ▸) *etc*. The experimental setup for a HERFD experiment is more involved than a standard XAS experiment since the technique requires ‘element-specific’ instrument optimization. In addition, for HERFD XAS applications a high-flux-density beamline is required, which limits biological applications to reasonably concentrated systems or those resistant to X-ray related damage.

### 3.2. Standardization of bio-XAS   

The expansion and success of bio-XAS is contingent on collaborative research that requires comparisons with published bio-XAS data. Since bio-XAS measurement and analysis techniques have grown organically over the past decades, it is increasingly difficult to directly compare two published bio-XAS data sets, to a point that research groups routinely measure samples that are already well documented in the published literature. In most cases, experienced research groups maintain an internal database of bio-XAS data, which have been prepared, measured and analysed using a known/preferred protocol. In fact, this is not limited to bio-XAS and is common practice for research groups in all areas of EXAFS application. In response to these database silos, EXAFS community members, individual researches and synchrotrons have created databases for free distribution of data, such as the Farrel Lytle database, the database hosted at XAFS.org, the SPring-8 data repository, actinide database, ESRF database for sulfur compounds and the more recent database created by the XAFS society of Japan. These databases have been, and continue to be, incredibly important to the community for qualitative comparison; however, quantitative comparison is difficult due to the lack of standard protocols for sample preparation and data collection.

A standard for data collection and analysis can address some of these barriers to quantitative comparison. The bio-XAS user community is the ideal test ground for the creation of such a standard. This is because specialized methods have been developed to navigate the complexities of biological samples and in doing so have led to the institution of standardized procedures for data measurement, analysis and interpretation. For example, bio-XAS beamlines the world over are equipped with specialized instrumentation such as liquid-He cryostats, ion chambers for reference compounds, 30- or 100-element Ge detector systems for fluorescence data measurement and automated in-hutch instrumentation that can control/minimize sample X-ray exposure. From a data analysis standpoint, since structural flexibility in biology limits the information content and precludes long-range order, EXAFS analysis is simplified. Therefore, in the overwhelming majority of biological EXAFS applications, the first shell is exploited for *quantitative* analysis and comparison, while the outer shells are treated in a more *qualitative* basis (barring longer metal–metal contributions). The main analytical thrust in bio-EXAFS publications rarely depends on the analysis of EXAFS outer shells. Additionally, since there is little hope of obtaining quantitative information from samples that have partial occupancies/incomplete loading or multiple different binding sites, successful bio-EXAFS studies are limited to homogeneous samples with complete occupancies. The result is that bio-EXAFS fits involve simple structural models with mostly single-scattering contributions and at most three-atom multiple-scattering contributions (in some biomimetic model complexes, four-atoms paths can also contribute significantly to the EXAFS data).

The following section presents a researcher’s perspective of bio-XAS data presentation and reporting, with the specific aim of creating a feedback loop for improving/standardizing data analysis protocols and optionally to make published data available to collaborators/researchers in a meaningful and quantitative format. Such a reporting standard, enforced by the user community and publishing groups alike, can be an important step towards standardization of data measurement and analysis techniques in bio-XAS.

## 4. Data reporting format   

The XAFS commission of IUCr has made significant effort towards standardizing the XAS data format. In 2012, under the auspices of the commission, a data standard format called the XAS data interchange format (XDI) was developed (Ravel *et al.*, 2012 ▸). The XDI has a plain text format with a header section that uses a clear yet succint syntax system to identify metadata represented in the following sections. The aim with XDI is to provide the minimum amount of information required to completely represent the experimental data obtained from a beamline at any synchrotron facility. If widely used, XDI can become a powerful format for data collection, sharing and analysis using tools currently available to the user community and will address several issues faced by the collective EXAFS community. Here, a data-reporting standard is proposed, which is aimed at improving data collection, analysis and reporting practices both for the novice and experienced bio-XAS researcher. The aim is to develop a XIF (XAFS Information File) file – an easily accessible text file which holds key information about the bio-XAS measurement and analysis. Journal enforcement (a XIF file will be the requirement for submission of a manuscript with bio-XAS data and analysis) and willingness to make it available as a supporting information document would be an ideal mechanism for widespread acceptance by the research community. Here, the focus in on the contents of the XIF file alone although the use of the ‘markdown’ format is advocated.

### 4.1. Data measurement consideration   

X-ray absorption spectroscopy gains strength from comparative analysis of unknown systems with known standards and this is especially true for bio-XAS. Thus, it is to the benefit of the novice bio-XAS researcher to closely follow literature protocols of data masurement, reduction and analysis if they want to make meaningful comparative analysis. For data measurement, this starts with the beamline resolution, energy calibration and following a consistent sampling protocol over the XAS spectrum, especially in the XANES to ensure that the spectral features in the pre-edge (1*s* → 3*d* transitions) and rising edge (contributions from 1*s* → 4*p* transitions, formally forbidden two-electron transitions and long-range multiple scattering) are adequately measured. Unlike the EXAFS region, the requirements for sampling in the XANES region is energy dependent and in some cases system dependent. In addition to the region file, the reference foil spectrum (where measured) can be of assistance to obtain sampling information, glitch patterns and a direct energy resolution comparison. Although the reference foil for energy calibration is the most widely used method for bio-XAS beamlines, it is not as accurate as absolute energy calibration methods developed, for example, by Pettifer & Hermes (1985 ▸). Here, the reference foil is used in the ‘Measurement block’ due to ease of reporting and its widespead use by the EXAFS community.

**Figure 4:**
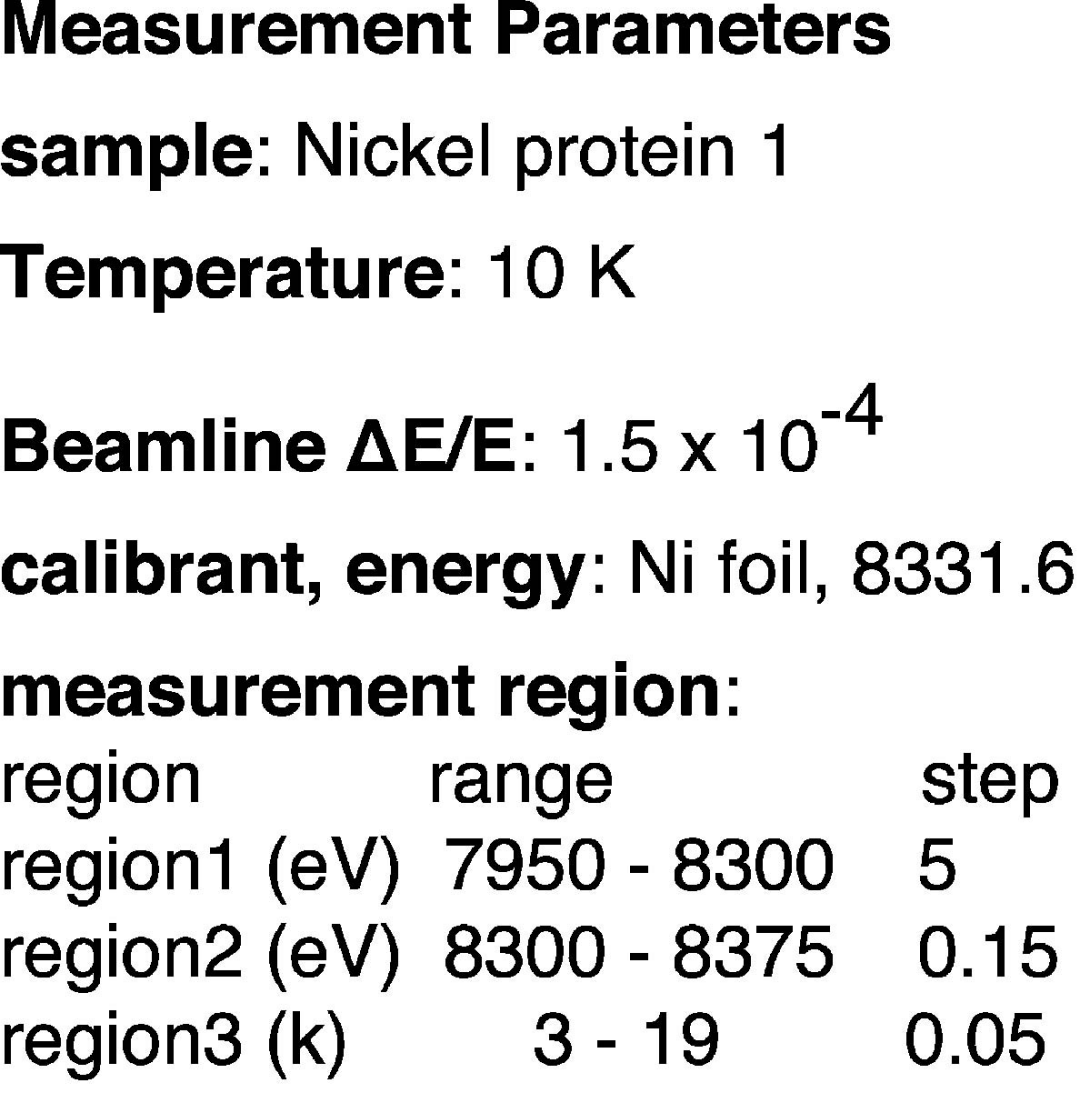
XAS data measurement parameters useful to the bio-XAS researcher for data measurement.

With this information, the first block, *i.e.* the ‘Measurement block’, of the XIF file can be created, which can help researchers obtain a bio-XAS spectrum directly comparable with literature data (Fig. 4 ▸). Note that the researcher is still responsible for ensuring and reporting on the sample quality and monitoring/alleviating photoreduction and artifacts that may arise from sample or instrument operation. This is already a widely accepted practice in bio-XAS reporting and publication. One other consideration for the ‘Measurement block’ is that it needs to be flexible for expansion for inclusion of more involved experiments, such as HERFD XANES and EXAFS. Since, the experimental section of the manuscript should include details of the spectrometer setup and alignment, an important parameter to include would be the energy position of emission peak maxima at which the HERFD XANES spectrum is obtained.

### 4.2. Data analysis considerations   

**Figure 5:**
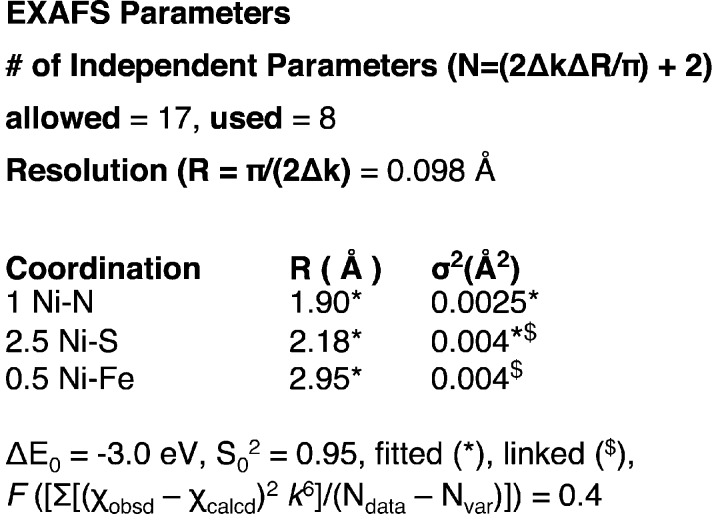
An example of the proposed EXAFS block for a typical bio-XAS measurement.

EXAFS data fitting is an iterative process that uses a trial-and-error approach of using theoretical phase and amplitude parameters from model structures (or a combination of several absorber–scatterer pairs) to fit the experimental data. These model structures are then refined to obtain better models and the process repeated until a satisfactory final-fit has been obtained. Depending on the scientific enquiry, the criteria to obtain and judge a ‘final fit’ can be vastly different. However, the best fit should follow cardinal rules such as adhering to the distance resolution and number of allowed independent parameters. Unfortunately, there are several literature examples which flout these rules. Ensuring the reporting of these parameters not only helps the researcher and reviewer to clearly evaluate the quality of the EXAFS fits but also underscores EXAFS good fitting practices for the existing and novice XAS community (Fig. 5 ▸). It is important to emphasize here that this information should be available in addition to standard data-fitting information presented in the experimental section. For a fit performed using *FEFF*, this includes parameters such as amplitude reduction factor, number of paths, distance (*R*), Δ*E*, σ^2^, cumulants related to thermal motion (where used), estimated standard deviations for various fitted parameters (where significant), *etc*. This includes the so-called ‘Hamilton test’, which can help determine the statistical significance of additional components in the EXAFS fit (Hamilton, 1965 ▸; Downward *et al.*, 2007 ▸).

With this information, the second block, *i.e.* the ‘EXAFS block’, of the XIF file can be created, which not only provides a succinct summary of the data-fitting protocol but also underscores the importance of often-ignored rules for EXAFS fitting to both the original author of the published study (creating a feedback loop for developing good data-fitting practices) and the new researcher interested in following the protocol in the original study.

### 4.3. Data sharing considerations   

**Figure 6:**
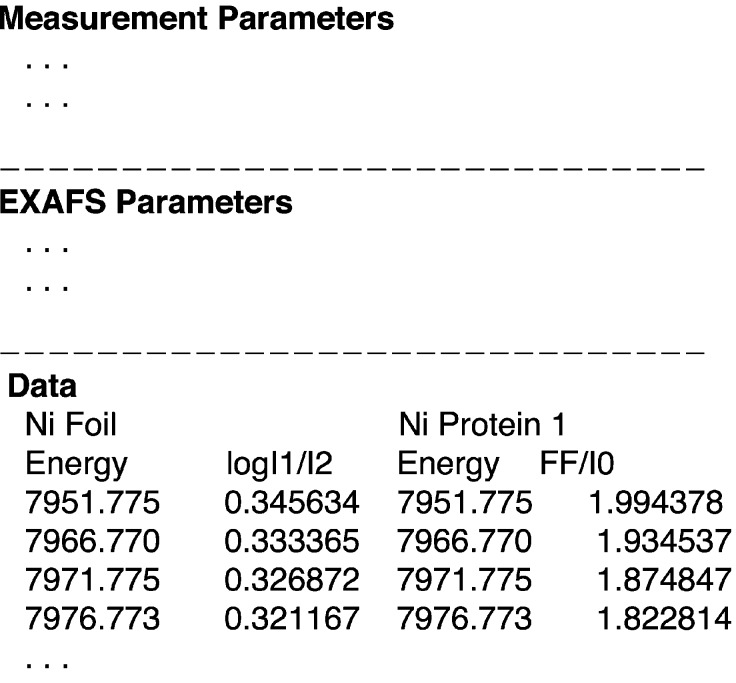
An example of the proposed bio-XAS XIF file with optional reference and protein fluorescence data column. The Measurement and EXAFS parameter blocks would have the content shown in Figs. 4 ▸ and 5 ▸, respectively. Only the first few data points of the optional data block are shown.

As mentioned in §4.1, a simultaneously measured reference foil spectrum holds key information about the beamline and experimental protocol. Addition of this spectrum to the XIF file would be invaluable in sharing and comparing data. In contrast to data on reference compounds, researchers feel strongly about the free availability of data on ‘real systems’ which is the subject of their investigation and the basis for new discoveries. However, national funding agencies worldwide are moving towards enforcing free availability of data, which will promote data sharing. Additionally, the availability of data digitization software means that access to published data is just a software operation away. It is therefore in the interest of the bio-XAS community to start sharing data on ‘real systems’. To achieve this, the XIF file can include a third optional ‘Data’ block. A representative bio-XAS XIF file, broken into blocks, *i.e.* Measurement, EXAFS and Data blocks, is shown in Fig. 6 ▸, wherein the Data block contains optional reference and protein data sets. The information presented in a XIF file that contains the data columns, along with the experimental protocol detailed in the main text of the publication, will be sufficient for any researcher in the field to at least reproduce the results presented in the publication, if not arrive at the exact same fit parameters.

## 5. Conclusion   

The data-reporting XIF file presented here can benefit the bio-XAS community in standardizing data measurement, reduction and analysis by helping develop a common methodology for data collection and demystifying EXAFS data analysis techniques by reporting fit-relevant parameters and emphasizing the limitations of information content in the data. The proposed XIF can be improved upon and generalized to include relevant blocks or parameters for other areas of XAS application. Since the bio-XAS community is comparatively small, the advantages and pitfalls of such a data-reporting format can be thoroughly tested with bio-XAS publications, before expansion to other areas. The success of any data-reporting format ultimately depends on journal cooperation, which can enforce the inclusion of a XIF file as a checklist item during the bio-XAS manuscript submission process. If useful to the general community, the XIF file could be developed as an output of popular data-fitting programs. The next logical steps should be the creation of a working group of bio-XAS members of IUCr to finalize the proposed XIF format and work with publishers for incorporation in the submission process.
